# Living with Food Allergy in Adolescence: The Nutritional and Psychological Impact of Allergen Avoidance

**DOI:** 10.3390/nu18010056

**Published:** 2025-12-23

**Authors:** Hanna Sikorska-Szaflik, Joanna Połomska, Barbara Sozańska

**Affiliations:** Department and Clinic of Paediatrics, Allergology and Cardiology, Wroclaw Medical University, ul. Chałubińskiego 2a, 50-368 Wrocław, Poland

**Keywords:** food allergy, adolescence, elimination diet, quality of life

## Abstract

The prevalence of food allergy continues to rise worldwide. The allergen profile of affected individuals changes with age: milk, egg, wheat, and soy allergies are more common in early childhood and often resolve before adolescence, whereas peanut, tree nut, fish, and shellfish allergies tend to persist and become more apparent in teenagers. The aim of this narrative review is to discuss the impact of food allergy and elimination diets on the nutritional status, psychological functioning, and quality of life of adolescents. Although an elimination diet remains the main way of treatment, it may lead to the risk of vitamin D, calcium, iron, and protein deficiencies, and potentially to growth disorders. In adolescents, irregular eating habits and poor adherence to dietary recommendations pose additional challenges. The psychological burden of living with food allergy include fear of accidental contact with the allergen, difficulties in peer relationships, and reduced quality of life, particularly in terms of social and emotional functioning. Lack of training in using an adrenaline autoinjector and embarrassment about its use are further risk factors in this age group. Optimal care for adolescents with food allergies requires a multidisciplinary approach, including an allergist, dietitian, and psychologist. Education, psychological support, and gradual development of self-management skills are crucial to improving the safety and quality of life of adolescents with food allergies. Further efforts by medical societies are warranted to advance the development of alternative therapeutic approaches for food allergy, including immunotherapy and biologic therapies, as well as to strengthen public health strategies for individuals affected by food allergies.

## 1. Introduction

Awareness of food allergy (FA) dates back many centuries. As early as 2700 BC, Chinese records recommended that pregnant women avoid certain foods believed to cause skin lesions. In the 1st century BC, Titus Lucretius Carus wrote “What is food to one, to another is rank poison”, clearly indicating that adverse reactions to food were already recognized at that time [1]. Recent advances in medical science, technology, and nutrition research have greatly improved our understanding of how certain foods can trigger harmful reactions in the body.

The prevalence of FA continues to rise worldwide. It is estimated to affect approximately 8% of children and 10% of adults in highly industrialized countries [2]. Significant regional differences are observed, and the reported prevalence varies depending on the specific food analyzed: in Europe and North America, peanut and egg allergies are the most common allergens, while in Asia, shellfish and fish allergy predominate [3]. Reactions to lipid transfer proteins (LTPs) in fruits, lentils, and walnuts are frequently reported in Southern Europe, whereas allergies to birch pollen and birch pollen cross-allergens (e.g., apple, carrot, celery, hazelnut) are common in Central and Northern Europe. Furthermore, the patient’s allergen profile changes with age: milk, egg, wheat, and soy allergies are more common in early childhood and often resolve before adolescence, whereas peanut, tree nut, fish, and shellfish allergies tend to persist and become more apparent in teenagers. Genetic, environmental, socioeconomic factors, and also dietary habits further contribute to the development and expression of FA [4].

Although new therapeutic options, including immunotherapy, are being developed, most patients still have to rely on elimination diets as the main or even the only effective strategy for managing their FA. Accidental exposures can occur even in the most vigilant patients and, although rarely fatal, they cause significant stress for both patients and their families. Elimination diets, particularly when multiple foods such as milk, eggs, or wheat must be avoided, may lead to nutritional deficiencies and growth retardation. FA also has an important psychological impact. Food-allergic children frequently experience anxiety, feeding difficulties, and social withdrawal, all of which may adversely impact the health-related quality of life (HRQoL) of affected children and their caregivers [5].

The aim of this narrative review is to examine the impact of FA and elimination diets on the nutritional status and psychological functioning of adolescents, as well as to discuss emerging perspectives in the management of food allergy. Particular attention is given to the treatment-related challenges, such as difficulties with adherence and reluctance to use adrenaline autoinjector due to embarrassment or social stigma. This review summarizes findings from studies identified through searches in PubMed, Google Scholar, and Web of Science, focusing on the years 2010–2025 and the terms “food allergy,” “adolescence,” “elimination diet,” and “quality of life.” The heterogeneity of study designs, populations, and methodological approaches may affect the extent to which the conclusions can be generalized.

## 2. Food Allergy: Mechanisms, Major Allergens, and Adolescent-Specific Characteristics

Adverse reactions to foods can be classified as immunological (allergic) or non-immunological (intolerances). Allergic hypersensitivity may occur as IgE-mediated (e.g., anaphylaxis), non-IgE-mediated, or mixed IgE and non-IgE-mediated conditions. In IgE-mediated FA patients produce antibodies against foods that non-allergic individuals tolerate without any problem. The mechanism is complex and involves the activation of certain immune cells like mast cell and basophil, their degranulation, with the release of mediators that can cause symptoms in various organs. Although immediate-type (IgE-mediated) hypersensitivity plays a key role in FA, cytotoxic and immune complex–mediated reactions may also contribute. The overall pathophysiology reflects a complex interaction between the intestinal mucosa, local and systemic immune reactivity, and the microbiome. Recent studies suggest that changes in the composition of the gut microbiome may influence both the development and persistence of FA [6,7,8]. Importantly, the risk of IgE-mediated food reactions is higher in children with a positive family history of allergic diseases. The clinical manifestations are heterogenous, vary among individuals, and depend on the underlying pathogenetic mechanisms triggered by the allergenic food. Symptoms may appear in different parts of the body, most commonly affecting the gastrointestinal tract, skin or respiratory system. However, in some patients, symptoms are limited to a single system. The onset of symptoms following allergen ingestion varies according to the underlying pathological mechanism, with reactions presenting immediately, after a delay, or at later stages [9,10].

Importantly, a clear distinction must be made between self-reported and physician-confirmed FA. Population-based studies show that the prevalence of self-reported FA significantly exceeds the prevalence of FA confirmed by objective diagnostic methods, including specific IgE testing and oral food challenge tests. This discrepancy may lead to an overestimation of the prevalence of FA and has significant clinical implications, such as unnecessary dietary restrictions and decreased HQoL, particularly during adolescence [11].

Certain foods are responsible for the majority of clinically significant IgE-mediated food allergic reactions worldwide. In the United States, nine of these foods (milk, eggs, fish, shellfish, peanuts, tree nuts, wheat, soy, and sesame) are legally defined as the “major food allergens,” or the Big Nine, which form the basis of allergen labeling regulations. This list was historically known as the “Big Eight” until the Food Allergy Safety, Treatment, Education, and Research (FASTER) Act of 2021 added sesame, and labeling requirements took effect in January 2023 [12].

During childhood, some food allergies naturally resolve, while others continue or new ones emerge. Early life is characterized by milk, egg, wheat, and soy allergies, which generally decline by the teen years. Meanwhile, allergies to peanuts, tree nuts, fish, and shellfish often remain or develop for the first time during adolescence [3]. This is reflected in the Isle of Wight birth cohort, where the prevalence of FA remained at approximately 5% in early childhood (ages 1–4), decreased to around 2.3% at age 10, suggesting partial resolution of early childhood allergies, and then increased again to about 4% at age 18, driven by new-onset allergies, particularly to peanuts, tree nuts, kiwi, and shellfish. These data indicate that adolescence may represent a period of renewed increase in the prevalence of food allergies or continued persistence in those who have not outgrown them [13].

In another cohort study of adolescents with FA followed for 15 months, participants experienced 34 allergic reactions per 100 patients per year, including 16.2 anaphylactic events per 100 patients per year. The study emphasized that foods such as peanuts, tree nuts, fish, and shellfish are particularly important allergens in this age group and pointed out that adolescence is a time when severe allergic reactions may occur more often [14].

The SchoolNuts study involved students aged 10–14 years from the Melbourne area who were suspected of having IgE-mediated FA. Both the students and their parents filled out questionnaires that asked about allergic reactions during the past 12 months—including their symptoms, triggers, and the situations in which they occurred. Almost half of the participants (44.4%) reported at least one allergic reaction to food in that time, and about one in ten (9.7%) had a reaction that met the criteria for anaphylaxis. Reactions most often occurred at home, with peanuts and tree nuts being the most common triggers. The risk of allergic reactions was higher in girls, in those with more than two food allergies, and in children with a history of asthma. Nut allergy was the only factor associated with anaphylaxis. Although reactions tended to be more severe in individuals with asthma, the association with the risk of anaphylaxis was not statistically significant. These results suggest that allergic reactions, including severe ones like anaphylaxis, occur quite often in early adolescence, particularly in those with multiple allergies, asthma, or nut allergy [15].

Beyond the physiological risks, adolescence represents a critical period of significant psychological, emotional, and social development, often accompanied by increased stress level and strong peer influence.

For teenagers with food allergies, the need to strictly avoid allergens, follow elimination diets, and carry emergency medications may further exacerbate these challenges. Lack of adherence to dietary and medical recommendations is common in this age group with only 16% of adolescents reporting full compliance with elimination diets and proper use of adrenaline autoinjectors. The requirement to stay constantly alert to avoid allergens, along with having to skip shared meals or refuse foods offered by friends, can increase anxiety and emotional stress, lowering overall HRQoL [16].

## 3. Elimination Diet and Nutritional Status

Although an elimination diet is the basis of FA therapy, it carries the risk of nutritional deficiencies, particularly during periods of rapid growth and adolescence. Some research indicates that children and adolescents who avoid food allergens—especially multiple allergens simultaneously—are more likely to experience deficiencies in key nutrients such as vitamin D, calcium, and iron, as well as lower height and weight gain compared to their peers [17,18].

In early childhood, eliminating common allergens, such as milk and wheat, is associated with a risk of nutritional deficiencies and mild growth retardation. In a prospective study, children avoided these allergens with dietary support, including specialized formulas; anthropometric and biochemical results were similar between groups, although mean height and weight remained slightly below population norms. Similar nutritional challenges have been observed in infants and toddlers, with around 35% showing deviations from normal growth and feeding difficulties strongly increasing the risk of malnutrition. The literature indicates that growth disturbances depend not only on the number of eliminated foods but are also associated with picky eating and feeding difficulties, which can further complicate nutrient intake. Even medically indicated elimination diets can negatively impact growth and nutrient absorption if not managed properly. This risk can persist into adolescence in individuals who need to follow long-term restrictive diets [19,20,21].

In a British prospective birth cohort study, children who had followed a cow’s milk exclusion diet in infancy were compared with control children aged 8–13 years. No differences in growth were observed between the groups; however, there was a trend toward a higher prevalence of overweight and obesity in the exclusion group, with nearly twice as many children classified as overweight or obese. These findings suggest that attention should focus not only on undernutrition but overweight may also be a problem [22].

In a cross-sectional study conducted in the UK, 81 adolescents (48 with confirmed FA and 33 controls) and 70 adults (23 FA and 47 controls) were assessed for dietary intake and anthropometric measures using a 4-day food diary and questionnaires. Among adolescents, those with FA had higher intakes of niacin and selenium compared with controls, even when supplements were not considered. The distribution of macronutrients also differed: adolescents with food allergies received slightly less energy from fat and more from carbohydrates. This pattern may reflect the avoidance of high-fat snacks that could contain allergens. Overall, total energy intake, protein intake, and BMI were similar between the groups. The authors concluded that dietary intake in food-allergic adolescents was generally similar or slightly better than in controls, but suboptimal intake of several micronutrients was observed in all participants. They suggested that these findings may be due to both intentional avoidance of allergen-containing foods and the fact that many nutrients are found in a wide variety of foods, which can make deficiencies less obvious [23].

According to the review by Di Cesare et al. the most frequently reported nutrient deficiencies in children on elimination diets due to FA are calcium and vitamin D, particularly among those avoiding dairy products. Other commonly observed deficiencies include zinc, iodine, vitamin B12, and folic acid. The authors presumed that these problems might result not only from avoiding certain foods but also from having a limited variety in the diet and insufficient nutritional guidance [24].

Systematic reviews indicate that children with multiple food allergies are at risk of nutritional deficiencies, such as lower levels of calcium, vitamin D, protein, and essential fatty acids. However, the impact varies depending on the number and type of allergens, age, and individual context. Nutritional counseling and regular dietetic follow-up have been shown to reduce this risk, improve nutrient intake, and support growth, highlighting the importance of personalized, age- and allergen-specific dietary management [25,26].

In the observational study, Papachristou et al. compared diet diversity, nutrient intake, and weight status between 100 children (aged 3 to 18) with IgE-mediated FA and 60 children with respiratory allergies as controls. The percentage of underweight children was higher in the FA group (19.6%) compared with controls (5.1%). Overall diet diversity was similar between groups (11–12 different foods/day), although children with FA consumed slightly more meat. Within the allergy group, children allergic to milk had lower protein and calcium intake, ate fewer commercially prepared sweets, and consumed more eggs compared with children with nut or egg allergies, yet diet diversity remained comparable. The authors underlined that, even when children with food allergies eat a varied diet, they can still be at higher risk of undernutrition. This is why early dietary guidance, provided just after diagnosis, is so important [27].

The population-based analysis of 6189 children (aged 2–17 years) from the USA examined the impact of FA on growth and nutrient intake using National Health and Nutrition Examination Survey (NHANES) data. Overall, 6.3% of children reported a FA, and milk was the most common trigger. Children with milk allergy had significantly lower height, weight, and BMI percentiles compared with nonallergic children, as well as smaller triceps skinfold measurements, indicating reduced adiposity. They also had lower calcium intake and tended toward lower vitamin D and total caloric intake. Importantly, these growth deficits were not fully explained by nutrient intake alone, suggesting additional mechanisms such as chronic allergic inflammation or increased metabolic demands. Other food allergies were associated with modest differences in nutrient intake but did not significantly affect growth measures. The findings highlight that children with milk allergy are at particular risk for nutritional compromise, supporting the need for close dietary supervision, supplementation when appropriate, and careful growth monitoring [28].

As some children naturally outgrow food allergies, their allergic status should be reassessed regularly to prevent unnecessary diets. It is known that treatment of FA often involves avoiding foods that contain nutrients essential for growth.

Adolescence is a critical period of rapid growth, maturation, and increasing nutritional needs, making dietary management for individuals with FA particularly challenging. Unlike younger children, adolescents assume greater responsibility for food choices while also exhibiting less dietary control, irregular eating habits, and a greater tendency to skip meals. In this context, FA-related food avoidance may disproportionately impact dietary quality and nutrient adequacy. Data on the nutritional consequences of FA in adolescents remain limited, as most available studies cover a wide age range of children rather than focusing exclusively on this stage of development. Consequently, conclusions regarding adolescents are often extrapolated from younger pediatric cohorts, a limitation that should be considered. However, despite the predominance of data from younger children, adolescents with FA may be at similar nutritional risk, given the increased nutrient requirements and the potential impact of prolonged food avoidance.

Recent data show that adolescents with FA often limit their food choices due to fear of accidental exposure rather than medical necessity. In a cross-sectional study of 46 adolescents, those with food allergies reported frequent anxiety about eating in restaurants, school cafeterias, friends’ homes, and while traveling [29]. These behaviors may contribute to reduced dietary diversity and selective eating patterns, emphasizing the need to assess nutritional status within the broader context of adolescents’ social functioning and perceived risk. Importantly, among individuals with FA, adolescents and young adults have been identified as the age group with the highest rate of fatal allergic reactions [30]. This vulnerability combined with increased nutrient requirements, greater autonomy in food choices, and less dietary supervision, highlighting the importance of research specifically targeting adolescents with FA.

## 4. Advances in Food Allergy Treatment

In recent years, food allergy treatment has been based not only on strict food avoidance but also on the search for therapeutic methods aimed at inducing desensitization or promoting long-term lack of reaction. Oral immunotherapy (OIT) is currently the most widely studied method. It involves the controlled administration of increasing doses of the allergen that is causing the patient’s allergy. OIT can lead to an increased reaction threshold (desensitization), primarily aiming to protect against reactions after accidental exposure. Although sustained immunological remission allowing dietary liberalization has been reported, this outcome remains uncommon and is not the primary goal of therapy [31]. At the same time, it should be remembered that although OIT may effectively induce desensitization, it significantly increases the frequency of allergic and anaphylactic reactions compared to allergen avoidance [32]. Sublingual immunotherapy and epicutaneous immunotherapy have lower risk profiles but also lower efficacy [33,34].

The development of biological therapies is important in the treatment of FA and its potential for the future. Omalizumab, an anti-IgE monoclonal antibody, significantly raises response thresholds to many allergens and may enhance the safety and efficacy of OIT used as adjunctive therapy [35]. Dupilumab, an IL-4/IL-13 receptor antagonist, has also been considered as a potential treatment option for food allergy. However, data from recent studies are somewhat conflicting: although Dupilumab is safe and reduces certain allergy biomarkers, it does not induce tolerance or desensitization to peanut and is not an effective [36].

The gut microbiota has been shown to play a key role in the development of oral tolerance and the pathogenesis of food allergies. Microbiome modification strategies through probiotics, dietary interventions, or other strategies represents a promising avenue for the prevention and treatment of food allergies, although further preclinical and clinical studies are necessary [37]. This underscores the need to shift the approach to food allergy from passive avoidance to proactive immune modulation. Incorporating these therapies into clinical practice may reduce the burden of strict elimination diets and improve the quality of life for affected patients.

While emerging therapies, including biologics and immunotherapy, offer promising approaches to modulate immune responses in FA, it is increasingly recognized that treatment alone may not be sufficient. Factors influencing immune development early in life, particularly nutrition and gut microbiota, play a critical role in shaping tolerance and allergy outcomes. Evidence from early allergen introduction studies, such as the LEAP and EAT trials, demonstrates that dietary exposures can actively promote oral tolerance and reduce the risk of IgE-mediated FA, especially to peanut and egg [38,39]. Moreover, specific dietary components, including long-chain polyunsaturated fatty acids, fiber-derived short-chain fatty acids (SCFAs), vitamins, and polyphenols, have been shown to modulate immune function by promoting regulatory T-cell responses and attenuating Th2-skewed inflammation [40].

Comprehensive approach to food allergy should integrate both therapeutic interventions and immune-modulatory strategies, including targeted nutrition, to optimize outcomes and potentially enhance the efficacy of emerging treatments.

## 5. Psychological Aspects of Life with Food Allergy

Children living with food allergies, as well as their families, often experience significant stress, anxiety, and lower HRQoL [41].

Food allergies affect more than just the body, they can also create emotional challenges and social difficulties, affecting both patients and caregivers. The cost of allergen-free products can add extra financial stress for families, especially for those with limited access to special foods or professional dietician support [19].

Fear associated with accidental exposure is one of the most commonly reported psychological responses. Mandell et al. observed that parents of children who had previously experienced food-induced anaphylaxis initially exhibited high levels of anxiety, which motivated them to seek information and implement effective management strategies [42]. Over time, this anxiety generally decreased, giving way to sustained vigilance. However, if anxiety levels declined too much, parental vigilance and preparedness also decreased, potentially increasing the risk of accidental exposure. Short-term anxiety triggered by events (such as exposure to new risks or developmental transitions) is considered adaptive, whereas chronic, paralyzing anxiety can interfere with effective coping [43]. Researchers call this the ‘Goldilocks principle’—just the right amount of worry helps children stay prepared without becoming overwhelmed [43]. In the context of FA the Goldilocks principle refers to the need to maintain an optimal balance between excessive allergen avoidance and unsafe exposure. In FA, this means eliminating only confirmed allergens while regularly assessing tolerance to avoid both allergic reactions and unnecessary dietary restrictions. This approach is particularly important for adolescents, whose autonomy and risky behaviors require balanced, individualized management strategies.

A study by Fedele et al. identified distinct patterns of adaptation among families of children with FA, differing in anxiety levels, management skills, and ability to integrate allergy care into daily life. They distinguished four profiles: Balanced Responders, High Responders, Low Responders, and Anxious High Responders with particularly elevated parental anxiety. The authors emphasized that both insufficient and excessive vigilance can impair family functioning and highlight the need for tailored psychological and educational support to promote balanced adaptation. Authors highlighted that both parental and child anxiety levels play a critical role in how families adapt to allergy-related problems [44]. Beyond the family context, social environments such as schools and peer interactions pose additional challenges. Studies indicate that children typically feel safe at home, where the environment is controlled. However, places like schools, restaurants, or during trips can make them anxious, as allergens are harder to predict and manage [41,43,45]. These circumstances can lead to avoidance of peer interactions, social events, or school trips, contributing to social isolation and limited participation in activities appropriate to child age.

Although children with FA do not always demonstrate higher levels of generalized anxiety compared with healthy peers, they often exhibit increased separation anxiety, panic symptoms, and anxiety related to potential adverse events, particularly in the absence of caregivers [43]. Anxiety disorders, reduced HRQL, and fear of serious allergic reactions have also been reported in adolescents with FA [46]. Shanahan et al. found that FA was associated with more symptoms of separation and generalized anxiety, ADHD, and, in addition, anorexia nervosa in a community-based epidemiological sample of adolescents [47].

According to a large population-based survey conducted in the United States, FA was found to be associated with increased school absenteeism. Sansweet et al. observed that over one-third of children with FA missed at least one school day because of allergy-related reasons, and that these absences were linked to greater psychosocial burden among affected families [48].

HRQL has been shown to be lower in children with FA, with significant reductions in social, emotional, and physical domains, particularly in older children and those with more severe allergies [41,43]. A history of anaphylaxis and the use of an adrenaline autoinjector were associated with higher levels of internalized psychological problems, emphasizing the impact of life-threatening allergic reactions on mental health [43]. Caregiver burden, particularly for mothers, is another important aspect, as elevated stress and anxiety, which in turn impacts family functioning and the child’s HRQoL [43,45]. Younger children depend on their caregivers to manage allergies, but as they grow older and begin to take more responsibility, they may experience both greater independence and greater anxiety, especially when apart from their parents. Parental anxiety and stress have been shown to predict children’s reported eating anxiety, social limitations, and the emotional impact of food allergies [43].

In a study conducted by Nguyen et al. adolescents, despite being allergic to fewer allergens, rated their HRQoL lower than younger children. This may be related to greater awareness of the disease, increasing independence in maintaining nutritional safety and less parental control. The authors mentioned that adolescents often experience greater emotional stress, with previous studies reporting symptoms such as depression, social isolation, and learning difficulties related to chronic illness. Furthermore, adolescents showed a greater sense of responsibility and understanding of the permanent nature of their condition, which may increase stress. The cited study also found that adolescents were less likely to experience fear of accidental allergen ingestion, and also that possessing an adrenaline autoinjector was associated with a greater sense of security. Other researchers suggest that quality of life declines with age in children with FA. Adolescents (13–17 years old) in the survey used by the authors report significantly higher overall scores than younger children (0–12 years old), reflecting greater emotional impact and more pronounced social and dietary limitations [49]. The results indicated that adolescents experience greater anxiety in situations such as eating in restaurants, at friends’ houses, or while traveling, reflecting a greater psychosocial burden compared to younger children. It also demonstrates that education and support in developing independence are crucial to improving HRQoL [50]. Similar patterns were observed in a Swedish study assessing FA-related HRQoL in adolescents (13–17 years) and adults (≥18 years) with confirmed allergy to milk, egg, or wheat. Both gender and age were found to influence HRQoL in this group. Men aged 18–39 years were at the greatest risk of deterioration, particularly in areas related to allergen avoidance and dietary restrictions, reflecting difficulties in coping with social situations and managing their diet independently. The authors suggest that these differences may be due to underdeveloped adaptive strategies and the complex transition from pediatric care to independent living. In women, HRQoL remained more stable regardless of age, which may indicate better adaptation to self-management and adherence to an elimination diet. Researchers underlined the need for clinical and educational support during adolescence and early adulthood for patients learning to manage their allergy independently [51].

The authors of the systematic review consider factors such as gender, age, disease severity, co-existing allergies and external influences, and examine how these factors may impact allergy-related quality of life and psychological distress, including anxiety and depression [52]. Subsequent studies have examined how specific demographic and clinical factors affect the daily functioning and well-being of adolescents living with food allergies. Socioeconomic status appears to influence HRQoL, partly through differences in access to allergen-free products, nutritional counseling, and specialist care. However, studies directly targeting adolescents remain scarce, and further research is needed to explore these effects across different healthcare and social systems [53].

The impact of gender on HRQoL in adolescents with FA is ambiguous. As mentioned above, higher psychosocial burden and poorer HRQoL were observed in boys and young men; however, in another studygirls showed clinically worse HRQoL than boys (mean difference 0.71), although this difference was only marginally statistically significant. The number of allergens and the type of symptoms, including a history of anaphylaxis, were not significantly associated with worse HRQoL. This study involved adolescents aged 13–17 years with FA, and the mean overall HRQoL was 4.70 on a scale of 1–7 (where 7 represents the worst HRQoL). The most affected domain was “allergen avoidance and dietary restrictions” [54]. Gender differences in the perception of HRQoL in FA likely depend on the specificity of the study group, the exact age of the participants, the degree of independence in allergy management, and other individual and environmental factors. Therefore, it is difficult to draw a clear conclusion regarding the role of gender in shaping the HRQoL of adolescents with FA.

Oral food challenge (OFC), the diagnostic gold standard for confirming FA and guiding dietary management, plays a key role in the care of children with FA. Available data show that both children who are actually allergic and those who ultimately do not develop allergies function as allergic individuals before the challenge—with similar levels of anxiety, uncertainty, and dietary and social restrictions. Only after the OFC an FA diagnosis can be definitively confirmed or ruled out, resulting in a significant improvement in HRQoL for most patients. In children with confirmed FA, the OFC results allow for the direction of further care and coping strategies, which is particularly important because the improvement in HRQoL visible after 2 months tends to diminish after 6 months. This requires ongoing monitoring and regular reinforcement of recommendations, as well as education to support families in maintaining established coping strategies [55]. Data from an analyzed cohort (0–17 years) confirm the overall safety of OFC, with low reaction rates and no serious events in the group of patients without a clear history of exposure. Importantly, age was not a factor influencing OFC results, indicating that both younger children and adolescents have a comparable risk of a positive or negative result [56]. Fear and anxiety connected with eating allergenic foods are common barriers to dietary reintroduction after OFC in children and adolescents [57]. This may reduce acceptance of OFCs and limit effective dietary liberalization following testing. Given the developmental context of adolescence, factors including autonomy in decision-making, concern about peer opinion, and heightened risk perception may further influence teens’ attitudes toward OFCs and highlight the need for tailored communication and support strategies in this age group.

Chronic stress related to adherence and risk of anaphylaxis is common. Families must balance strict adherence to dietary recommendations with the fear of life-threatening reactions, which can maintain ongoing stress [42,43]. Psychological interventions, including psychoeducation, counseling, relaxation techniques, and cognitive-behavioral therapy, have been shown to improve coping, reduce clinically significant anxiety, and improve psychosocial well-being in both children and caregivers [43]. FA affects not only physical health but also emotional, social, and family functioning. Recognizing these psychological aspects and implementing supportive interventions are essential to optimize coping, promote adaptive functioning, and improve HRQoL in children with FA and their families. (Table 1)

## 6. Food-Induced Anaphylaxis Management in Adolescents

As adolescents strive for independence in managing their allergy, developing practical skills and emergency preparedness becomes a key element of safe self-management. Compared to younger children, the potential for prevention and disease modifying therapies is more limited in adolescents, whose immune system has lost much of the plasticity seen in early childhood and is therefore less responsive to immunomodulatory approaches. Therefore, the presence of FA in an adolescent, suggests that this health condition is likely to persist in subsequent years and continue into adulthood [58].

The clinical manifestations of IgE mediated food allergic reactions can range from mild symptoms to severe, potentially life threatening anaphylaxis. Consequently, this implies the necessity of preparing the adolescent for the possibility of experiencing a severe immediate hypersensitivity reaction triggered by food. Anaphylaxis occurs in approximately 0.05% to 2% of individuals in the United States and around 3% in Europe, with numerous studies noting a growing number of cases [59,60].

In studies conducted by Oriel et al., the most common setting for adult food allergic reactions was the home, with restaurants identified as the second most frequent location. In children the opposite phenomenon was observed in the study. The authors demonstrated that allergic reactions in restaurants occurred even when the staff had been notified about FA, when the allergen was listed in the menu and even in situations where both measures were taken. Reactions were less commonly reported in school and in the workplace, representing 6% of cases among children and 11% among adults, respectively. Children who needed two doses of adrenaline had an average age of 11 years, those who were hospitalized over 14 years, and the average age of children requiring Intensive Care Unit care was 8 years [61].

The findings highlight the importance of establishing standardized protocols for all such settings, aimed at preventing and managing allergic reactions. It is particularly important in cases involving underage patients who experience anaphylaxis without the supervision of a legal guardian.

It is essential that patients are educated to identify which of the symptoms they experience are in fact allergic reactions, to evaluate the severity of the symptoms, and understand the appropriate steps to take in response.

According to The European Academy of Allergy and Clinical Immunology (EAACI) guidelines on the management of IgE mediated FA a personalized medical treatment plan should be prepared [62].

As a medical plan intended for the patient, the management strategy should be adapted to the patient’s age and level of responsibility. For young children, the plan is primarily directed at parents and other adult caregivers, while adolescents are expected to manage their condition more independently.

Therefore it should be written in language understandable to the adolescent and include contact information for trusted adults and local emergency medical services.

A key component of the patient’s medical treatment plan is a comprehensive list of food allergens that must be strictly avoided. This necessitates a thorough discussion of potential hidden sources of these allergens—not only with parents or caregivers but also with adolescents, who may independently choose, purchase and consume food products.

As a part of this process, both the adolescent and caregivers should receive education on how to read food labels, recognize alternative names for allergens, and identify high -risk food categories.

The majority of fatal food induced anaphylactic reactions happen in individuals already diagnosed with FA, despite many having only experienced non-severe reactions in the past [60].

This knowledge is critical to minimizing accidental exposures and promoting safe, age-appropriate self-management.

Adolescence is marked by increased impulsivity, with decisions often guided more by emotions and social influences than by rational thinking [63]. Potential consequences include an increased propensity for risk-taking behaviors and the use of psychoactive substances, including alcohol [64]. Simultaneously, alcohol consumption may heighten risk-taking behaviors, such as consuming allergenic foods or meals of unknown composition without taking prior precautions. Alcohol can influence the course of an allergic reaction and lower the threshold for its onset, and is considered a classic cofactor in food-induced allergic reactions [65]. Therefore it is essential to educate adolescents about the effects of psychoactive substance use on the risk of triggering allergic reactions to food.

In addition to managing their condition, adolescents often become educators within their social circles. It is believed that risk-taking behavior may contribute to the elevated risk of fatal food-induced anaphylaxis in adolescents and young adults [66,67].

They must be prepared to refuse unsafe foods, even when this was met with misunderstanding or social pressure.

Crealey et al. studied Irish children (2–16) with confirmed FA as a part of prospective observational study. Nearly half of participants (41.5%) brought their own food to social events, and 38% never ate food prepared at friends’ houses. The results highlight that their guardians perceive accidental ingestion—not environmental contact—as a primary risk, and that bringing food from home helps children participate socially in a safe way [68].

The financial burden of purchasing allergen free-products falls more heavily on families with fewer resources and less access to support [69].

When an adrenaline autoinjector or inhaled medication is part of the medical management plan, patient education should ensure that the adolescent is able to use these treatments correctly and independently in an emergency situation.

This includes hands-on training, regular practice with trainer devices and simulation of emergency scenarios.

A study by Noimark et al. involving children treated in pediatric allergy clinics across the United Kingdom, aimed to evaluate how frequently adrenaline autoinjectors were used during anaphylactic episodes and to explore the reasons for their omission when administration was clinically warranted. The findings revealed that only a small proportion of children experiencing anaphylaxis in community settings received adrenaline. The most frequently cited reasons for not using the autoinjector included the perception that it was not needed (54.4%) and uncertainty about whether its use was appropriate (19.1%) [67].

Support from healthcare providers is important in building adolescent competence and confidence in real-life application [62].

The indications for prescribing an adrenaline autoinjector in IgE mediated FA are divided into absolute and relative indications, according to the current EAACI Anaphylaxis Guidelines. Prior anaphylactic episodes caused by food allergens, asthma that is uncontrolled or classified as moderate to severe persistent, presence of systemic mastocytosis as an underlying condition are among the indications for the prescription of self-injectable adrenaline [62]. More research is needed to improve adherence to adrenaline use when anaphylaxis occurs.

## 7. Discussion

FA in adolescence represents a complex interaction of nutritional, psychological, and social factors. This developmental period is characterized by increasing autonomy, the evolution of risk-taking behaviors, and increasing responsibility for health decisions, which can negatively impact adherence to allergen avoidance strategies. Consequently, adolescents with FA deal with unique challenges that differ from those experienced by younger children.

**Figure 1:**
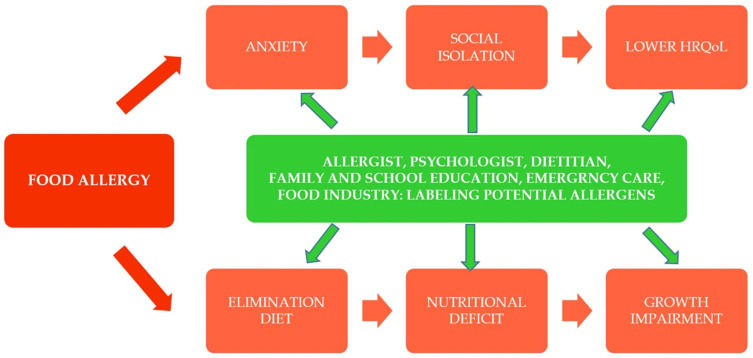
Model illustrating the relationships between FA, elimination diet, psychological functioning, and HRQoL, emphasizing the need for multidisciplinary care.

A multidisciplinary approach involving allergist, dietitian, psychologist, and school nurse is crucial in coordinating the care of the patients with FA and their family. This approach enables comprehensive medical and psychosocial support, addressing the daily challenges faced by adolescents with food allergies (Figure 1).

Moreover, it is worth considering implementing educational programs in primary healthcare, schools, and patient organizations to support the transition from pediatric to adult care. These programs could include practical training in label reading, emergency procedures, and the use of epinephrine autoinjectors, as well as standardized care plans tailored to the patient’s age, fostering independence and self-confidence. Their effectiveness should be systematically assessed, particularly in the context of dietary adherence, anxiety reduction, and readiness to use epinephrine when necessary.

Educational programs implemented in schools and preschools have so far focused primarily on training staff in allergen avoidance, anaphylaxis recognition, and prompt use of adrenaline autoinjector. Observational studies have shown that such interventions resulted in improved knowledge and preparedness for emergency situations [70].

However, training and preparedness are not uniform across all settings. A cross-sectional survey of teachers in Saudi Arabia, found that although most teachers were aware of FA and could recognize anaphylaxis, only 15.4% knew that epinephrine is the first-line treatment, and nearly 40% of schools lacked a formal action plan for managing food-allergic students [71]. These findings highlight the need for standardized educational strategies and school policies to ensure effective management of food allergies in educational settings. Age-appropriate educational interventions have also been found to increase awareness and reduce stigma toward peers with FA [72]. Nevertheless, evidence of long-term effectiveness, particularly regarding anxiety reduction and dietary adherence, is limited.

Research indicates that both excessive and insufficient parental vigilance negatively impacts adolescents’ confidence and HRQoL. Therefore, training programs could also incorporate elements of psychological support and family therapy, aimed at improving HRQoL, reducing anxiety, and developing rational vigilance and shared responsibility between parents and children.

According to a recent systematic review, combining educational interventions with standard medical treatment plays an important role in improving the HRQoL of adolescents with FA and their parents [73]. The review analyzed various forms of support, including psychological interventions, handbook provision, cognitive-behavioral therapy, mentorship, and practice-based programs. Among these, supportive and educational approaches showed the most favorable outcomes. The authors emphasized that developing structured, accessible, and sustainable support interventions should remain a key goal in clinical practice to enhance overall care and HRQoL [73].

Because many allergic reactions occur at home, in restaurants, and at schools, it is important to standardize management of care across educational settings, including staff training, providing access to epinephrine, and provision of allergen-free meals in school cafeterias. These actions may potentially reduce the frequency of allergic adverse reactions and reduce anxiety levels in patients and their families.

Given the specific nature of adolescence, it is also worth developing the use of digital tools and peer support initiatives as ways to foster independence. Mobile applications for monitoring nutrients, notification systems, online educational platforms, and online and in-person peer support groups can facilitate daily functioning and reduce feelings of social isolation.

## 8. Unmet Needs

Despite growing recognition of adolescence as a critical period in FA management, several unmet needs remain. Data specific to adolescents are limited, as many studies include children across different age groups without addressing this developmental stage. As a result, evidence addressing the unique nutritional, psychological, and social challenges experienced by adolescents is insufficient. There is a need for long-term, representative studies following adolescents with FA from childhood through adolescence into adulthood. These studies should assess the impact of increasing independence, changes in risky behaviors, and educational interventions on long-term health outcomes and HRQoL. Such research would provide a better understanding of factors promoting safety, adherence, and psychological well-being during the transition to adulthood. Previous studies (e.g., [15,50,51,54]) indicate that adolescence is an important stage of development, but data tracking the long-term consequences of these changes are still lacking. Standardizing research methodology and reporting methods would also be important to facilitate comparisons and the possibility to create meta-analyses in future research.

## 9. Limitations of the Study

It should be noted that the available literature is largely limited to studies including wide pediatric age ranges rather than focusing specifically on adolescents. Consequently, conclusions regarding adolescents are often extrapolated from pediatric cohorts, which may not fully reflect the unique nutritional, psychological, and social challenges of adolescence. Additionally, heterogeneity in study design, outcome measures, and HRQoL assessment tools limits direct comparison between studies and precludes robust meta-analyses.

## 10. Future Directions

FA and anaphylaxis pose a significant public health challenge due to their prevalence, potential severity, and impact on adolescents’ HRQoL. Strategies to minimize these effects include early diagnosis, patient and family education, psychosocial support, access to epinephrine, innovative treatments such as immunotherapy and biologics, and transparent food labeling. Educational programs in schools and other public settings are crucial, emphasizing staff training in allergen avoidance, anaphylaxis recognition, basic life support, and proper epinephrine autoinjector use. Legal frameworks for epinephrine administration vary by country. Regional differences also exist in access to treatment and the implementation of educational initiatives, particularly between developed and developing countries. Patient associations and the allergy community can contribute to this development by establishing guidelines and standards, promoting research. They may also support public and school-based educational programs, often in collaboration with government or non-governmental organizations to increase awareness, preparedness and patient safety. Future research should prioritize long-term, prospective studies following individuals with FA; furthermore, it is essential to implement broad-based, multi-level interventions that combine medical, nutritional, psychological, and social interventions. Regularly assessing the effectiveness of these programs, flexibly responding to new challenges, and adapting them to the individual needs of young people are essential to truly improve the health and HRQoL of patients with FA.

## Figures and Tables

**Table 1:** Psychosocial consequences of FA affecting HRQoL in adolescents [19,41,42,43,44,45,46,47,48,49,50,51,52,53,54].

Domain	Psychosocial Consequences	Possible Manifestation
Emotional functioning	increased anxiety fear of accidental exposure decreased sense of safety	avoidance of social events involving food preoccupation with checking ingredients anticipatory anxiety before meals eaten outside home
Social functioning	social withdrawal difficulties with maintaining peer relationships feelings of isolation	refusing invitations to meetings or school trips embarrassment when discussing dietary restrictions perceived stigma
Self-perception	low self-esteem feeling “different” from peers	negative body image due to dietary restrictions reluctance to use an adrenaline autoinjector in public
School environment	stress related to eating at school reduced concentration avoidance of canteens	limited participation in shared meals or school events increased vigilance leading to distraction in class increased school absenteeism
Family relationships	parental overprotection family tension conflicts about diet adherence	restrictive household rules anxiety during family meals disagreements about safety and independence

## Data Availability

Not applicable.
